# Serum lipid reference values recommended during a twin pregnancy and evaluating its association with perinatal outcomes

**DOI:** 10.1186/s12884-023-06110-6

**Published:** 2024-01-02

**Authors:** Jiaqi Huang, Xinlu Meng, Jiaxin LI, Xiaoli Gong, Tianchen Wu, Huifeng Shi, Xiaona Li, Xueju Wang, Jing Yang, Yangyu Zhao, Yuan Wei

**Affiliations:** Peking University Third Hospital, Beijing, China

**Keywords:** Blood lipids, Reference range, Twin pregnancy, Pregnancy outcomes

## Abstract

**Background:**

Maternal lipid metabolism fluctuations have been shown to increase the risk of adverse pregnancy outcomes. However, there is no consensus over what constitutes normal maternal lipid values during twin pregnancy. Therefore, the aim of this study was to establish a serum lipid reference range for a twin pregnancy.

**Methods:**

A retrospective survey was conducted, from 2011 to 2021, at the Peking University Third Hospital. A total of 881 twin pregnancies, with lipid data from early and middle pregnancies, were included. After excluding those with adverse pregnancy outcomes, we performed a descriptive analysis of total cholesterol (TC), triglycerides (TG), high-density lipid cholesterol (HDL-C), and low-density lipid cholesterol (LDL-C) levels, using the mean and standard deviation to determine appropriate percentiles. We later determined the lipid reference range in early and middle pregnancy based on the initial results. We evaluated Inappropriate lipid levels associations with pregnancy outcomes, including gestational diabetes, pregnancy-induced hypertension, small for gestational age.

**Results:**

(1) Serum levels of TC, TG, LDL-C, and HDL-C increased significantly from early to late pregnancy, where the greatest increase was observed in TG. (2) Based on the results, we recommend that TC, TG, and LDL-C serum reference values during early and middle pregnancy should be less than the 95th percentile. On the other hand, HDL-C should be greater than the 5th percentile. During early pregnancy, the values recommended are TC < 5.31 mmol/L, TG < 2.25 mmol/L, HDL > 1.02 mmol/L and LDL < 3.27 mmol/L, and those during middle pregnancy are TC < 8.74 mmol/L, TG < 4.89 mmol/L, HDL > 1.25 mmol/L and LDL < 5.49 mmol/L, while the values during late pregnancy are TC < 9.11 mmol/L, TG < 6.70 mmol/L, HDL > 1.10 mmol/L and LDL < 5.81 mmol/L. Higher levels of blood lipids were associated with GDM, PE, SGA.

**Conclusions:**

We suggested a reference ranges for blood lipids during the twin pregnancy in a Chinese population. The reference ranges recommended by this study can be used to identify women with twin pregnancies using unfavorable lipid values. Higher levels of blood lipids were associated with adverse pregnancy outcomes.

## Background

Blood-based lipids include neutral fats (cholesterol CH, triglyceride TG) and lipoids. Plasma lipoprotein is a spherical macromolecular complex that consists of apolipoprotein and neutral fat. Plasma lipoproteins include chylomicron (CM), very low-density lipoprotein (VLDL), low-density lipoprotein (LDL), high-density lipoprotein (HDL), and lipoprotein(a) (Lp(a)) [1]. Cholesterol is not only an important component of biofilm but also a precursor of various steroid hormones. Cholesterol can activate the Sonic Hedgehog protein to transmit development signals for different systems, such as neural tube, brain, heart, and limbs, after the embryo implantation [2].

Triglycerides, the energy source with the largest production capacity and reserves in the body, provide energy for the growth and development of the fetus [3]. Plasma lipoproteins are not only important carriers for cholesterol transport; for instance, low-density lipoprotein is also the key precursor for progesterone synthesis by the corpus luteum, which plays a crucial role in the maintenance of pregnancy in the first trimester [4].

Pregnancy is a unique physiological phase of life for women, where adaptive physiological and metabolic changes occur to ensure the needs of fetal growth and development, during which lipid metabolism undergo significant changes [5]. Maternal dietary intake increases in the first and second trimesters of pregnancy, while insulin resistance heightens fatty acid synthesis. At the same time, lipoprotein lipase (LPL) activity in adipose tissues also picks up, causing the acylation of free fatty acids into triglycerides for storage [5]. In the third trimester of pregnancy, insulin resistance and LPL activity of adipose tissues decrease, such that the anabolism of adipose tissues in the first trimester of pregnancy turns into a net decomposition of the maternal adipose reserve by the second trimester of pregnancy, which results in a net increase in blood lipid levels [6]. Studies have shown that abnormal blood lipid changes during pregnancy may lead to complications such as preeclampsia, premature delivery, and gestational diabetes mellitus, causing adverse pregnancy outcomes [7]. In recent years, an extensive application of assisted reproductive technology and ovulation induction drugs has significantly increased the incidence of twin pregnancy. Compared with singleton pregnancy, twin pregnancy is a high-risk pregnancy, and is more prone to bring about complications such as high blood pressure, high blood glucose, low weight, and asphyxia, thereby causing some adverse pregnancy outcomes, and affecting the safety of mothers and fetuses [8]. Studies have shown that the normal range of blood lipids in non-pregnant adults is not suitable for pregnant women and that the blood lipid levels in women with a twin pregnancy are higher than those with a single pregnancy [9]. Hence, understanding the normal range of blood lipids in women during the early, middle, and late stages of a twin pregnancy would further help strengthen the management of women with a twin pregnancy and thereby help reduce the incidence of adverse pregnancy outcomes. At present, there are very few studies on the diagnosis and management of blood lipid levels during pregnancy, with no international consensus regarding its management. At present, Williams Obstetrics (24) and Beijing and Guangzhou, respectively, have proposed the reference range of blood lipids during the second trimester of a single pregnancy for people living in Europe, America, and northern and southern China [10]. The Williams Obstetrics recommendations for serum lipids value during first pregnancy are TC 3.65–5.44 mmol/L, TG 0.45–1.8 mmol/L, HDL1.04–2.02 mmol/L and LDL1.55–3.96 mmol/L, and those during middle pregnancy are TC4.56–7.74 mmol/L, TG 0.85–4.32 mmol/L, HDL1.35–2.25 mmol/L and LDL1.99–4.77 mmol/L, while the values during late pregnancy are TC5.67–9.04 mmol/L, TG1.48–5.12 mmol/L, HDL1.24–2.25 mmol/L and LDL 2.62–5.80 mmol/L [11]. However, the changes in blood lipid levels and their reference range during twin pregnancies have not been explored yet. The purpose of this study, therefore, was to investigate the blood lipid levels in women with a twin pregnancy at our hospital.

## Methods

### Data sources

A retrospective survey was conducted, total of 881 women with twin pregnancy, without any complications, were investigated and delivered in the Third Peking University Medical Obstetric Hospital, from January 2011 to December 2021. Patients who met one of the following conditions were excluded: ① Chronic hypertension; ② Type 1 and type 2 diabetes mellitus; ③ Thyroid disease, immune system disease and other serious systemic diseases or chronic consumptive diseases; ④ Previous dyslipidemia, and ⑤ Inadequate data on important items (blood lipid data during pregnancy, pregnancy outcome, age, etc.).

### Clinical data collection

The clinical data of enrolled patients were collected using the medical record system at the Third Hospital of Peking University, including age, pregnancy frequency, delivery time, mode of delivery, adverse events, outcomes of pregnancy, and blood lipid levels during pregnancy. The blood lipid values of all patients were recorded at the biochemical laboratory of our hospital. Triglyceride (TG) levels were determined by the enzyme method, and total cholesterol (TC) was determined by the cholesterol oxidase method. High-density lipoprotein (HDL) and low-density lipoprotein (LDL) were determined by the homogeneous phase method. The average reference value intervals of blood lipids in early, middle, and late pregnancy, and the average blood lipid levels every 4 weeks were calculated. Changes in the blood lipid levels during the pregnancy were also observed.

### Statistical analysis

The SPSS26.0 software was used for statistical analysis. The results of continuous variables were described by mean standard deviation, and an independent sample t-test or a one-way ANOVA was used to compare the differences among continuous variables between groups. *P* < 0.05 indicated the point of differences being statistical significance. Hospital lipid thresholds were calculated using the 2.5 or the 97.5 centiles. TC, TG and LDL above the 97.5th centile were abnormal, while HDL below the 5th centile was abnormal.

## Results

A total of 881 women with twin pregnancies were enrolled in the study. Of them, 75.3% were appropriate aged, and 24.7% were over aged. BMI was defined according to the WHO Asia Pacific BMI criteria [12], 64.2% of the group were of standard weight, while 13.5% were underweight, 18.9% were overweight, and 3.4% were obese. 85.1% of the children were first born. Among them, 68% were dichorionic diamniotic (DCDA) twins, and 32% were monochorionic-diamniotic (MCDA) twins, as no monochorionic-monoamniotic (MCMA) twins were observed (Table 1).

**Table1:** Meternal baselin characteristics

	[x^−^ ± s, n(%)]
Age(year)	31.92 ± 3.68
p-BMI(kg/m^2)	21.83 ± 3.08
18.5	119(13.5)
18.5–24	563(64.2)
24–28	166(18.9)
≥ 28	30(3.4)
Gravidity	1.61 ± 1.13
Parity	0.16 ± 0.39
Type	
DCDA	596(68)
MCDA	280(32)

**Fig. 1:**
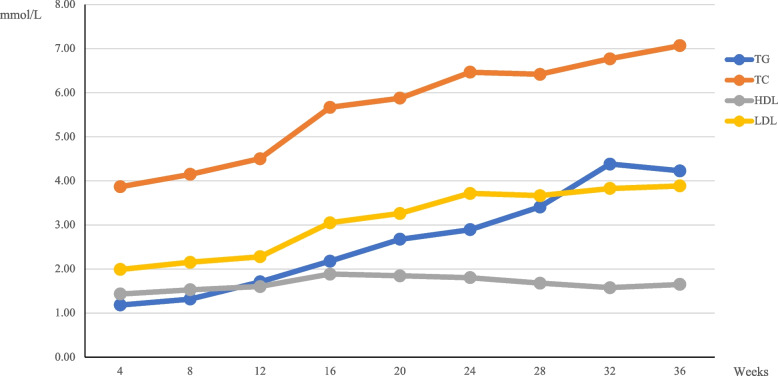
Maternal blood lipid levels during pregnancy (Abbreviations: TC, total cholesterol; TG, triglycerides; HDL-C, high-density lipid cholesterol; LDL-C, low-density lipid cholesterol)

The average values and centiles of TC, TG, HDL, and LDL during the early, middle, and late pregnancy are shown in Table 1. With increasing gestational weeks and pregnancy periods, TC, TG, HDL, and LDL showed an overall increasing trend, among which TG showed the most significant change, which are shown in Fig. 1. The TG level during late pregnancy was 3.29 times the value during early pregnancy. In addition, the levels of TC, LDL, and HDL during late pregnancy were 1.67, 1.12, and 1.81 times higher than the values during early pregnancy, respectively.

According to the 5th or 95th centile, the reference range for blood lipids was established (Table 2). The values were as follows; First trimester: TC 2.87–5.39 mmol/L, TG 1.26–2.27 mmol/L, HDL 1.01–2.02 mmol/L and LDL 1.17–3.29 mmol/L; Second trimester: TC 4.64–5.00 mmol/L, TG 1.43–2.27 mmol/L, HDL 1.22–2.60 mmol/L and LDL 1.93–5.55 mmol/L; Third trimester: TC 4.56–9.23 mmol/L, TG 1.91–6.71 mmol/L, HDL 1.08–2.60 mmol/L, and LDL 1.92–5.93 mmol/L.

**Table 2:** Serum lipids in twin pregnant women

	trimester	x¯ ± s	Percentile										
			2.5%	3%	5%	10%	25%	50%	75%	90%	95%	97%	97.5%
	First (*n* = 357)	4.01 ± 0.64	2.87	2.92	3.06	3.22	3.60	3.94	4.43	4.94	5.15	5.31	5.39
TC	Second (*n* = 188)	6.36 ± 1.05	4.64	4.72	4.85	5.14	5.57	6.25	7.10	7.82	8.43	8.74	8.78
	Third (*n* = 245)	6.71 ± 1.18	4.56	4.94	5.04	5.19	5.81	6.57	7.48	8.31	8.88	9.11	9.23
	First (*n* = 358)	1.26 ± 0.44	0.52	0.54	0.62	0.73	0.93	1.20	1.53	1.87	2.16	2.25	2.27
TG	Second (*n* = 186)	2.86 ± 0.94	1.43	1.48	1.60	1.75	2.26	2.76	3.43	3.97	4.46	4.89	5.00
	Third (*n* = 241)	4.15 ± 1.19	1.91	2.01	2.29	2.69	3.28	4.05	4.95	5.77	6.36	6.70	6.71
	First (*n* = 359)	2.11 ± 0.53	1.17	1.22	1.33	1.49	1.76	2.06	2.45	2.84	3.11	3.27	3.29
LDL	Second (*n* = 185)	3.60 ± 0.87	1.93	2.00	2.19	2.56	3.01	3.53	4.11	4.80	5.21	5.49	5.55
	Third (n = 247)	3.81 ± 1.02	1.92	2.03	2.19	2.61	3.07	3.73	4.45	5.23	5.63	5.81	5.93
	First (*n* = 358)	1.48 ± 0.26	1.01	1.02	1.08	1.15	1.29	1.49	1.64	1.81	1.96	2.01	2.02
HDL	Second (*n* = 186)	1.83 ± 0.34	1.22	1.25	1.33	1.41	1.59	1.80	2.03	2.28	2.48	2.58	2.60
	Third (*n* = 245)	1.66 ± 0.32	1.08	1.10	1.12	1.24	1.42	1.65	1.86	2.06	2.24	2.32	2.35

**Fig. 2:**
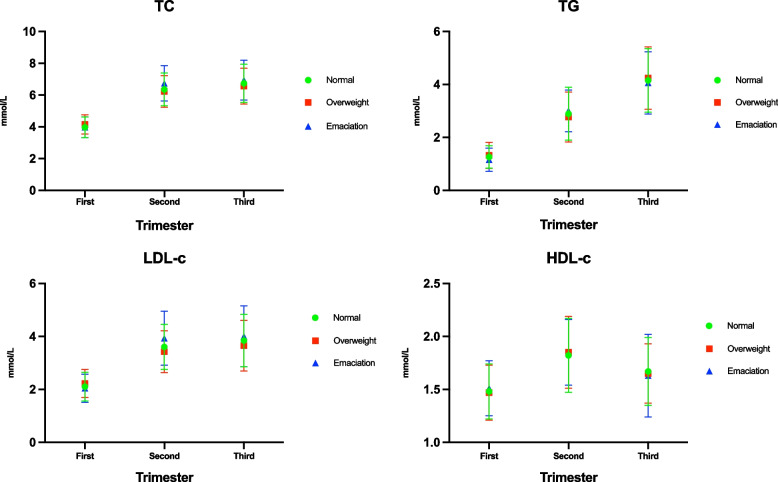
Comparison between different groups of maternal blood lipids, based on BMI, during pregnancy (Abbreviations: TC, total cholesterol; TG, triglycerides; HDL-C, high-density lipid cholesterol; LDL-C, low-density lipid cholesterol)

The patients were classified into emaciation, normal and overweight groups according to their BMI values. The normal values of blood lipid levels during each pregnancy were calculated and compared among the groups so that any statistical differences between the different BMI groups were eliminated (Fig. 2).

**Fig. 3:**
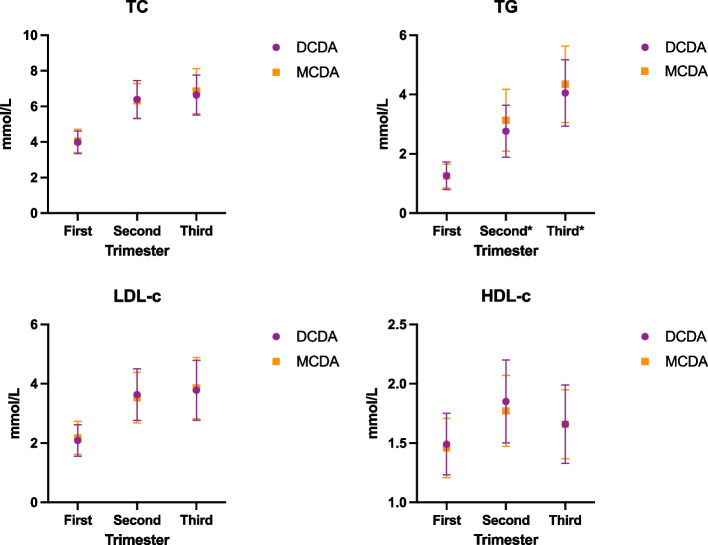
Comparison of maternal blood lipid levels between different villous membrane groups during pregnancy (Abbreviations: DCDA, Double chorionic double amniotic sac; MCDA, monochorionic double amniotic sac; TC, total cholesterol; TG, triglycerides; HDL-C, high-density lipid cholesterol; LDL-C, low-density lipid cholesterol)

The patients were divided into the DCDA group and MCDA group according to their villous membrane capacity, and the normal values of blood lipid levels in each pregnancy were calculated and compared among the groups. Among the groups based on villous membrane capacity, there were significant differences in triglycerides only during the second and third trimester of pregnancy (Ps = 0.0135, Pt = 0274), which was significantly higher in the MDCA group than in the DCDA group (Fig. 3).

**Fig. 4:**
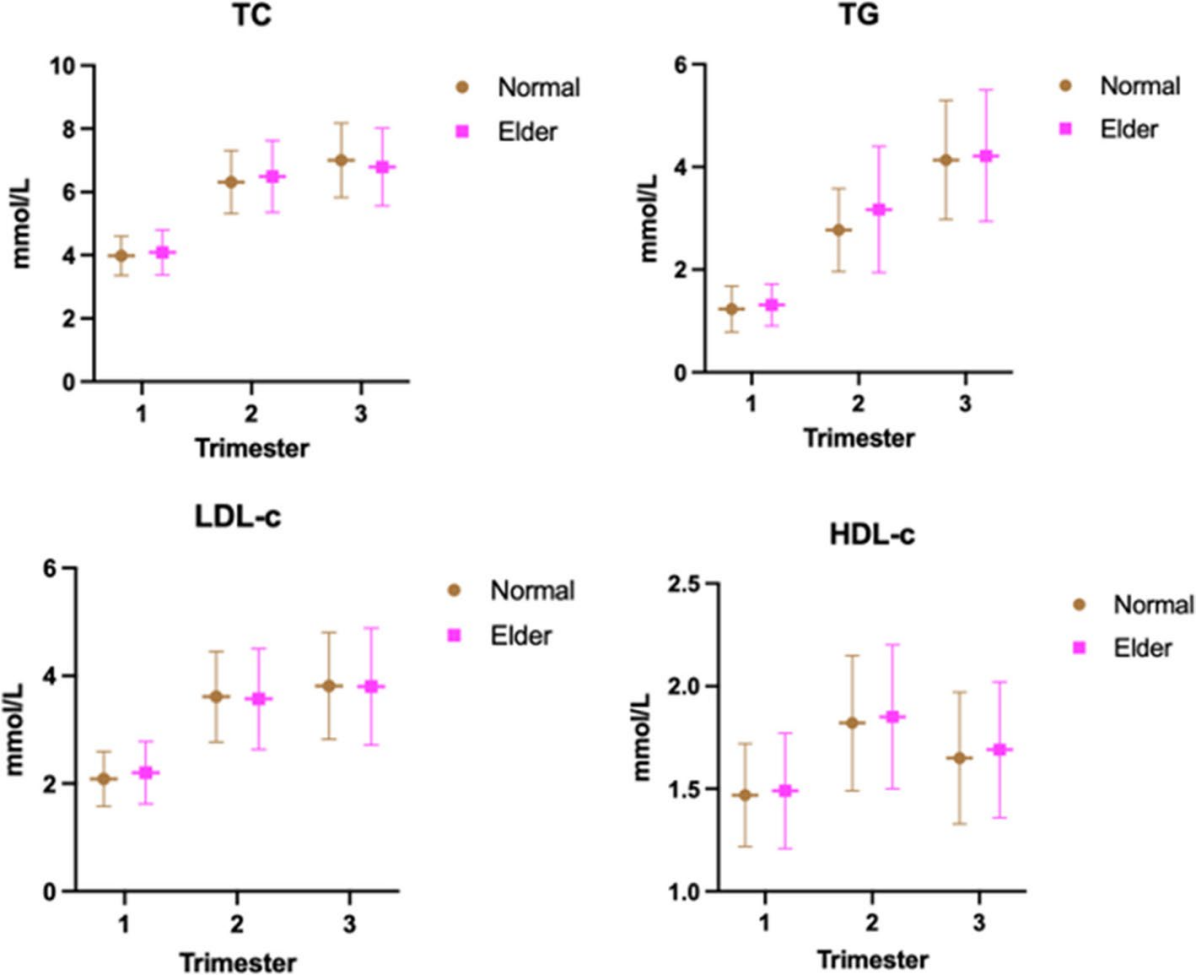
Comparison of maternal blood lipid levels between different age groups during pregnancy (Abbreviations: TC, total cholesterol; TG, triglycerides; HDL-C, high-density lipid cholesterol; LDL-C, low-density lipid cholesterol)

The subjects were divided into normal-age and older groups based on whether they were older than 35 years or not. The normal values of blood lipid levels in each pregnancy were calculated, and the comparison between groups showed that there was a statistical difference between different the age groups, but only in the case of triglyceride in the second trimester (*P* = 0.0117), while the blood lipid levels in the older group were significantly higher than the age-appropriate group (Fig. 4).

Tables 3, 4 and 5 show the perinatal outcomes associated with blood lipid in twin pregnancy in different trimesters. In the first trimester, higher levels of TCHO, TG, LDL-c, and lower HDL-c level were associated with increased risk of GDM, PE, SGA and sIUGR. In each trimesters, higher levels of TG were associated with increased risk of GDM.

**Table 3:** comparison of perinatal outcomes in women with higher blood lipids levels in the first trimester

	TCHO		TG		LDL-c		HDL-c	
	*P*	OR	*P*	OR	*P*	OR	*P*	OR
GDM	0.022*	1.734(1.083–2.779)	0.005*	1.934(1.220–3.065)	0.009*	1.898(1.170–3.080)	0.028*	2.857(1.117–7.309)
PE	0.436	0.707(0.296–1.690)	0.027*	2.048(1.083–3.871)	0.088	0.357(0.109–1.165)	0.595	0.576(0.075–4.402)
SGA	0.264	1.313(0.814–2.116)	0.007*	2.020(1.217–3.352)	0.644	0.894(0.556–1.438)	0.386	1.586(0.559–4.500)
sIUGR	0.882	1.070(0.440–2.598)	0.159	1.731(0.807–3.712)	0.915	0.949(0.364–2.474)	0.866	0.839(0.109–6.444)

*Values are given as *P* < 0.05

*Abbreviations*: *GDM* getational diabetes mellitus, *HDL-c* high-density lipoprotein cholesterol, *LDL-c* low-density lipopotein cholesterol, *OR* odds ratio, *SGA* small for gestional age, *sIUGR* selective intrauterine growth restriction, *T* total cholesterol, *TG* triglycerides

**Table 4:** comparison of perinatal outcomes in women with higher blood lipids levels in the second trimester

	TCHO		TG		LDL-c		HDL-c	
	*P*	OR	*P*	OR	*P*	OR	*P*	OR
GDM	0.219	0.777(0.347–1.742)	0.000*	2.626(1.626–4.238)	0.799	0.467(0.218–1.001)	0.49	1.761(0.953–3.253)
PE	0.476	1.255(0.365–4.322)	0.104	1.404(0.649–3.038)	0.561	1.176(0.399–3.463)	0.978	1.751(0.698–4.394)
SGA	0.442	0.856(0.389–1.884)	0.145	1.493(0.869–2.567)	0.896	0.977(0.493–1.939)	0.464	1.908(0.897–4.060)
sIUGR	0.804	0.934(0.316–2.761)	0.486	1.125(0.588–2.152)	0.000*	2.686(1.305–5.528)	0.701	1.087(0.467–2.530)

*Values are given as *P* < 0.05

*Abbreviations*: *GDM* getational diabetes mellitus, *HDL-c* high-density lipoprotein cholesterol, *LDL-c* low-density lipopotein cholesterol, *OR* odds ratio, *SGA* small for gestional age, *sIUGR* selective intrauterine growth restriction, *TC* total cholesterol, *TG* triglycerides

**Table 5:** comparison of perinatal outcomes in women with higher blood lipids levels in the third trimester

	TCHO		TG		LDL-c		HDL-c	
	*P*	OR	*P*	OR	*P*	OR	*P*	OR
GDM	0.512	1.138(1.083–2.779)	0.003*	1.999(1.252–3.192)	0.144	1.037(0.496–2.165)	0.633	1.065(0.451–2.515)
PE	0.824	0.847(0.251–2.857)	0.392	1.119(0.527–2.376)	0.877	1.231(0.418–3.623)	0.779	1.299(0.376–4.486)
SGA	0.276	0.671(0.340–1.324)	0.863	1.097(0.676–1.778)	0.275	0.562(0.283–1.116)	0.958	0.978(0.424–2.253)
sIUGR	0.340	1.483(0.595–3.699)	0.313	0.707(0.326–1.532)	0.914	1.217(0.456–3.247)	0.741	1.396(0.465–4.190)

*Values are given as *P* < 0.05

*Abbreviations*: *GDM* getational diabetes mellitus, *HDL-c* high-density lipoprotein cholestrol, *LDL-c* low-density lipopotein cholesterol, *OR* odds ratio, *SGA* small for gestional age, *sIUGR* selective intrauterine growth restriction, *TC* total cholesterol, *TG* triglycerides

## Discussion

Blood lipid levels increased during the first to the third trimester in twin pregnant women. In this study, we analyzed the blood lipid profiles of women with a twin pregnancy at our hospital during each pregnancy period and explored the reference value intervals of blood lipid levels during each pregnancy period. The results demonstrated that serum lipid levels during pregnancy were all significantly increased, with the most significant increase in TG and the smaller increases in TC, LDL, and HDL. The 5% reference value range has been a general reference calculation method in medicine. According to the 5th or 95th centile, the reference value of blood lipids was established during early pregnancy: TC < 5.31 mmol/L, TG < 2.25 mmol/L, HDL > 1.02 mmol/L and LDL < 3.27 mmol/L; Second trimester: TC < 8.74 mmol/L, TG < 4.89 mmol/L, HDL > 1.25 mmol/L and LDL < 5.49 mmol/L; and Late pregnancy: TC < 9.11 mmol/L, TG < 6.70 mmol/L, HDL > 1.10 mmol/L and LDL < 5.81 mmol/L. The reference range of blood lipid in non-pregnant adults in our hospital was as follows: TC < 5.18 mmol/L, TG < 1.7 mmol/L, HDL > 1.04 mmol/L, and LDL < 3.64 mmol/L. As compared to non-pregnant adults, women with twin pregnancies have markedly elevated blood lipid levels during pregnancy, especially during the second and third trimesters. Although there were no significant differences in mean blood lipid levels during the first and second trimesters of pregnancy when compared with blood lipid levels in singly pregnant women in China. Reference values established based on the 5th, or 95th percentile showed significant differences in total cholesterol, triglycerides, and low-density lipoprotein cholesterol in the second trimester.

Many articles have reported that the blood lipid level during pregnancy was elevated, where the most significant change was noticed in triglycerides [4, 13, 14], which was consistent with the results of this study. Changes in blood lipid levels during pregnancy were associated with the changes, including increased maternal appetite, intake, and insulin sensitivity during the early and middle stages of pregnancy, all of which promote lipid synthesis [5]. The increase in insulin sensitivity activates fatty acid synthesis in adipocytes and the expression of lipoprotein lipase [15]. In addition, substances like progesterone, leptin, and prolactin [16], also heighten fat storage. Late pregnancy is the second stage of lipid metabolism, which is the catabolism stage, in which insulin resistance and the increase of placental lactogens both enhance the lipolysis of triglycerides in adipose cells and synthetic lipoprotein in the liver, due to which overall lipid levels are increased [4]. Studies have also shown that blood lipid levels recover to pre-pregnancy levels post-delivery, indicating that increased blood lipid levels during pregnancy play an important role in the process and, thereby, have a critical role in the physiological development of the fetus [16].

Dyslipidemia during pregnancy can have adverse fetal and maternal consequences, like any other metabolic abnormalities. Like many other studies, we have substantiated that during the first and the second trimester of pregnancy, the incidence of adverse pregnancy outcomes tends to increase with an increase in total cholesterol, triglycerides, and low-density lipoprotein or with a decrease in high-density lipoprotein [4]. Especial, the triglyceride levels during early pregnancy had a relationship with increased risks such as gestational diabetes, preeclampsia, and small for gestational age. All the results upper suggesting that women in their childbearing age ought to control their blood lipid levels during pregnancy or even before pregnancy.

In this study we found that elevated blood lipid profiles may increase the risk of SGA. Elevated maternal blood lipid levels imply excessive lipid transport across the placenta to the fetus. Growth-restricted fetuses showed reduced lipoprotein lipase activity [17]. A low level of lipoprotein lipase has been associated with the impaired development of adipose tissue and augmented circulating TG levels [17].

In addition, we found that elevated LDL-c profiles may increase the risk of sIUGR. There may also be other unexplored mechanisms to explain the effect of elevated lipid levels in late pregnancy on maternal and fetal health. Further studies regarding the mechanisms are needed.

At present, adult blood lipid standards are still utilized in clinical studies. However, as per the results of this study, the blood lipid levels in pregnant women with twin pregnancy were significantly increased during pregnancy, compared with non-pregnant adults. Obstetricians could not determine whether the blood lipid levels in pregnant women were normal and thus could not determine whether blood lipid control through diet and exercise was mandated to reduce adverse pregnancy outcomes.

The results of this study provided useful reference data for blood lipid level management in pregnant women, even though this study was a single-center retrospective study with a small sample size. Therefore, the results of this study need to be further verified in future prospective multi-center studies with larger, representative sample sizes. Second, this exploratory study evaluated the associations between blood lipid levels from the first to the third trimester and multiple pregnancy outcomes. We identified some associations, whereas interpretation of these results should be made with caution. Further studies are needed to confirm the associations.

However, our study still has some limitations. First, retrospective studies might have limitations due to potential confounding factors, such as educational status, alcohol consumption, and smoking status. Information on pre-pregnant lipids levels is also insufficient. Second, potential selection bias may be present because our institution is a tertiary care center. Data from multicenter studies are needed to validate the conclusions of this study.

In summary, we found that the overall lipid levels showed an increasing trend during pregnancy. The results of this study suggested that a specific reference range of blood lipid levels during twin pregnancy should be established. The critical blood lipid levels during different stages of pregnancy recommended in this study were as follows: TC < 5.31 mmol/L, TG < 2.25 mmol/L, HDL > 1.02 mmol/L and LDL < 3.27 mmol/L during early pregnancy; TC < 8.74 mmol/L, TG < 4.89 mmol/L, HDL > 1.25 mmol/L and LDL < 5.49 mmol/L during the second trimester of pregnancy; and TC < 9.11 mmol/L, TG < 6.70 mmol/L, HDL > 1.10 mmol/L and LDL < 5.81 mmol/L, during late pregnancy. We also identified that dysbiosis of lipid metabolism is associated with increased risk of SGA and sIUGR. These findings suggested the complex association of lipid metabolism with fetal growth and development.

## Data Availability

The datasets generated analysed during the current study are not publicly available due the unpolished article, but are available from the corresponding author on reasonable request.
